# The Mutation of Conservative Asp268 Residue in the Peptidoglycan-Associated Domain of the OmpA Protein Affects Multiple *Acinetobacter baumannii* Virulence Characteristics

**DOI:** 10.3390/molecules24101972

**Published:** 2019-05-22

**Authors:** Jūratė Skerniškytė, Emilija Karazijaitė, Julien Deschamps, Renatas Krasauskas, Romain Briandet, Edita Sužiedėlienė

**Affiliations:** 1 Institute of Biosciences, Life Sciences Center, Vilnius University, Saulėtekio ave. 7, LT-10257 Vilnius, Lithuania; e.karazijaite@gmail.com (E.K.); renatas.krasauskas@gf.vu.lt (R.K.); edita.suziedeliene@gf.vu.lt (E.S.); 2 Micalis Institute, INRA, AgroParisTech, Université Paris-Saclay, 78350 Jouy-en-Josas, France; julien.deschamps@inra.fr (J.D.); romain.briandet@inra.fr (R.B.)

**Keywords:** *Acinetobacter baumannii*, OmpA, peptidoglycan, virulence, outer membrane vesicles, β-lactams

## Abstract

*Acinetobacter baumannii* is a nosocomial human pathogen of increasing concern due to its multidrug resistance profile. The outer membrane protein A (OmpA) is an abundant bacterial cell surface component involved in *A. baumannii* pathogenesis. It has been shown that the C-terminal domain of OmpA is located in the periplasm and non-covalently associates with the peptidoglycan layer via two conserved amino acids, thereby anchoring OmpA to the cell wall. Here, we investigated the role of one of the respective residues, D268 in OmpA of *A. baumannii* clinical strain Ab_169_, on its virulence characteristics by complementing the Δ*ompA* mutant with the plasmid-borne *ompA*_D268A_ allele. We show that while restoring the impaired biofilm formation of the Δ*ompA* strain, the Ab_169_*ompA*_D268A_ mutant tended to form bacterial filaments, indicating the abnormalities in cell division. Moreover, the Ab_169_ OmpA D268-mediated association to peptidoglycan was required for the manifestation of twitching motility, desiccation resistance, serum-induced killing, adhesion to epithelial cells and virulence in a nematode infection model, although it was dispensable for the uptake of β-lactam antibiotics by outer membrane vesicles. Overall, the results of this study demonstrate that the OmpA C-terminal domain-mediated association to peptidoglycan is critical for a number of virulent properties displayed by *A. baumannii* outside and within the host.

## 1. Introduction

The spread of multidrug-resistant (MDR) bacterial pathogens is of increasing concern [1,2,3]. *Acinetobacter baumannii* demonstrates the highest resistance rates among hospital-associated MDR bacteria during the last 10 years [4]. *A. baumannii* can cause ventilator-associated pneumonia, soft tissue, bloodstream and catheter-associated urinary tract infections; however, its virulence characteristics are still largely unknown [5].

Bacterial outer membrane proteins act as crucial factors in cell-to-cell signaling, adhesion and environment sensing, as well as in the protection against host immunity [6,7]. *A. baumannii* outer membrane protein A (OmpA) (~38 kDa) is a highly abundant outer membrane protein [8]. Experimental data characterize the OmpA protein as a multifunctional virulence factor, participating in *A. baumannii* biofilm formation, adhesion to epithelial cells, inhibition of host immune-response and resistance to various antimicrobial drugs [9,10,11,12].

The structure of OmpA is characterized through the N-terminal domain of the β-barrel structure, which is localized in the bacterial outer membrane and C-terminal OmpA-like domain, which allocates in a periplasmic space [13,14]. The OmpA protein interacts with the bacterial peptidoglycan and can mediate its interaction with the bacterial outer membrane, thereby maintaining its integrity [13,15]. Moreover, it has been suggested that the interaction of the OmpA protein with peptidoglycan may contribute to the production of outer membrane vesicles (OMVs), which are linked to *A. baumanni* pathogenesis [16].

Two conservative amino acids: D271 and R286, in the C-terminal OmpA-like domain of *A. baumannii* OmpA were identified by comparison of OmpA-like proteins from various human pathogens [17]. These residues have been shown to be critical for the non-covalent association of OmpA to diaminopimelate amino acid, a component of *A. baumannii* peptidoglycan, as demonstrated by the isothermal titration calorimetry using purified recombinant OmpA proteins with D271A and R286A substitutions, respectively [17].

While the involvement of the transmembrane domain of the *A. baumannii* OmpA protein in the transport of β-lactam antibiotics has been proposed [8,14], the role of C-terminal peptidoglycan-associated domain in pathogenesis remains unclear. Recently, the contribution of the C-terminus of the OmpA-like domain in resistance of the *A. baumannii* strain ATCC 17978 to several antibiotics was demonstrated [18]. Thus, other *A. baumannii* virulent features may also be dependent on the functionality of the periplasmic peptidoglycan-associated OmpA-like domain.

The aim of the present study was to investigate the role of association of the OmpA protein to peptidoglycan on the virulence characteristics of the *A. baumannii* clinical strain Ab_169_, its Δ*ompA* mutant and the *ompA*_D268A_ complemented strain, carrying substitution of one of the key residues required for OmpA interaction with the peptidoglycan.

## 2. Results

### 2.1. Effect of OmpA D268A Substitution on A. baumannii Biofilm Morphology

To investigate the role of the periplasmic OmpA C-terminal domain (OmpA-like domain) on the *A. baumannii* virulence characteristics, a clinical MDR strain Ab_169_ (Table S1) was chosen. Ab_169_ belongs to a common sequence type ST231 of widespread international clonal lineage I (IC I). The conservative residues, shown to be important for OmpA association to peptidoglycan [17], correspond to D268 and R283 residues in the Ab_169_ OmpA amino acid sequence. The Ab_169_ *ompA* gene and its variant with D268A substitution were cloned into a plasmid pUC_gm_AciORI (Table S1) resulting in plasmids p*ompA* and p*ompA*_D268A_. The plasmids were introduced into the Ab_169_Δ*ompA* mutant strain, obtained as described in the Materials and Methods section. OmpA production in generated strains was confirmed by SDS-PAGE gel and Western blot analysis (Figure S1).

**Figure 1:**
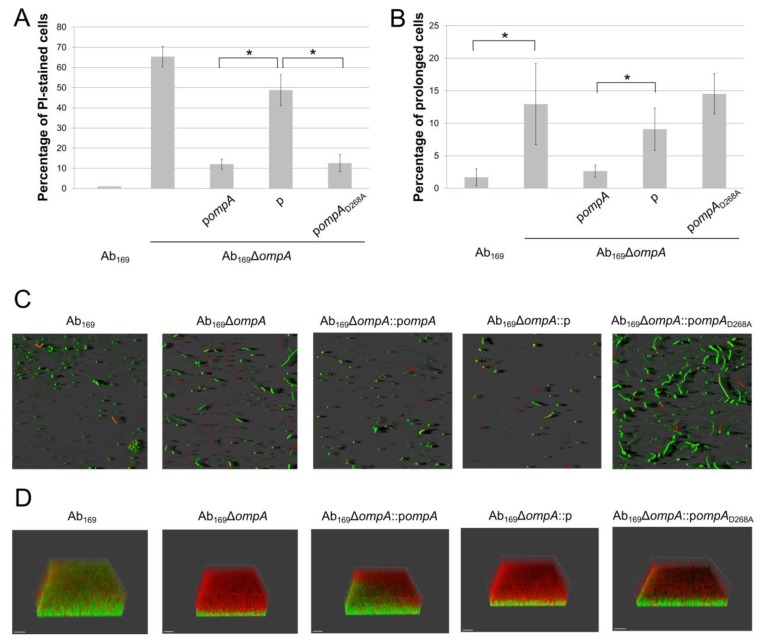
Confocal laser scanning microscopy (CLSM) analysis of *Acinetobacter baumannii* biofilms formation. (**A**) percentage of propidium iodide-stained bacteria after 2 h of incubation, compared with the total amount of cells. (**B**) percentage of prolonged cells after 2 h of incubation, compared with the total amount of cells; error bars represent standard deviations from six measurements of six different CLSM pictures, significance was assessed by *t*-test, (* *p* < 0.05). (**C**) visualization of initial attachment to the plastic by *A. baumannii* strains assessed after 2 h of incubation; bacteria were stained with SYTO9 (green) and propidium iodide (red). (**D**) three dimensional (3D) projections of mature biofilm formation after 24 h of incubation.

First, we analyzed the biofilm forming capacity of Ab_169_ and the Ab_169_Δ*ompA* mutant, complemented with a control plasmid and with plasmids p*ompA* or p*ompA*_D268A_. The strains were tested for their initial attachment to the plastic by incubating in Luria–Bertani (LB) medium at 37 °C for 2 h. The biofilm analysis was undertaken by confocal laser scanning microscopy (CLSM). As can be seen in Figure 1A, two hours after seeding, most of the Ab_169_ cells, attached to the plastic were viable, as judged from the dominance of SYTO9 stained cells (green color). The *ompA* gene knockout in Ab_169_ resulted in an approximately 65% increase in the amount of propidium iodide (PI; red color) stained cells. Interestingly, colony-forming units (CFUs) counting revealed only up to a five-fold decrease of viable cells in biofilm formed by Ab_169_Δ*ompA* compared with its parent strain. Therefore, PI-stained cells could indicate the dead cells as well as cells with the reduced cell wall integrity as a consequence of OmpA loss, albeit still maintaining viability. Additionally, the Δ*ompA* mutant tended to form prolonged bacterial chains (Figure 1B,C), most likely due to the impairment of cell division, as has been demonstrated in other bacteria with impaired peptidoglycan maintenance [19,20,21]. The Ab_169_Δ*ompA* complementation with a p*ompA* plasmid resulted in a significantly lowered number of PI-stained cells and an absence of prolonged bacterial cells chains (Figure 1A–C), whereas in the Ab_169_Δ*ompA* cells with a control plasmid, this phenotype was clearly visible. The introduction of a plasmid-borne *ompA* allele with D268A substitution into the Ab_169_Δ*ompA* strain efficiently reduced the number of PI-stained cells, although was not able to eliminate the phenotype of prolonged cell chains (Figure 1A–C).

After 24 h of incubation, *A. baumannii* mature biofilm structures were examined (Figure 1D). The PI-stained cells were found to be distributed mostly on the top of biofilm formed by the Ab_169_ strain. In contrast, the biofilm of the Ab_169_Δ*ompA* mutant contained a substantially increased amount of PI-stained cells, and prolonged cell chains were also evident. Both of these phenotypes were largely eliminated by the introduction of a p*ompA* plasmid. Similarly, the Ab_169_Δ*ompA* strain complementation with the plasmid carrying an *ompA*_D268A_ allele also resulted in a reduced amount of PI-stained cells in a mature biofilm, however, it did not eliminate the phenotype of prolonged cell chains (Figure 1D).

Overall, the observations described above imply that while the interaction of the OmpA C-terminal domain with the peptidoglycan was not critical in supporting biofilm formation, it was required for a proper *A. baumannii* cell division.

### 2.2. OmpA D268A Substitution Reduces A. baumannii Motility and Resistance to Desiccation

Next, we investigated *A. baumannii* features, which are important for its survival in a hospital environment. It was observed that alterations in peptidoglycan synthesis affect virulence-associated phenotypes, such as biofilm formation and the motility of bacterial pathogen *Campylobacter jejuni* [22]. Similar impairments in bacterial motility were observed in a strain of gut symbiont *Burkholderia* with altered peptidoglycan synthesis [23]. Twitching motility is a common feature of clinical *A. baumannii* strains [24], therefore, we investigated the role of OmpA association with peptidoglycan on the manifestation of this characteristic.

**Figure 2:**
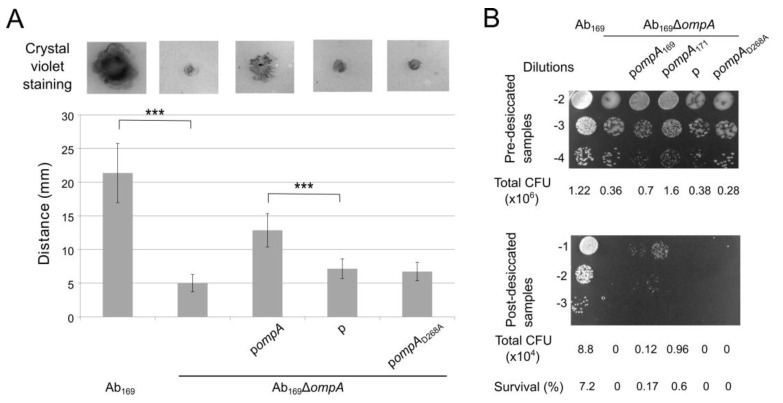
Twitching motility and desiccation resistance of *A. baumannii.* (**A**) twitching motility was determined using crystal violet staining, demonstrating the twitching zone in mm of motile strains; error bars represent standard deviations from at least three independent experiments; significance was assessed by *t*-test (*** *p* < 0.001). (**B**) resistance to desiccation; 10-fold dilutions of pre-desiccated and post-desiccated samples were seeded on Luria–Bertani (LB) plates; *ompA* gene from Ab_171_ strain belonging to IC II was used as an additional control.

The Ab_169_Δ*ompA* strain showed a loss of twitching motility compared with the parent strain, as evident from an absence of the colony expansion zone in between the LB agar and plastic surface interface (Figure 2A). The complementation with the *ompA* allele present on the plasmid restored the phenotype, although not to the full extent, whereas an empty plasmid was not able to rescue the phenotype. However, no complementation was seen upon the introduction of plasmid with the *ompA*_D268A_ gene variant, thereby indicating that OmpA association with peptidoglycan is needed for a display of this type of motility in *A. baumannii*.

The ability to withstand long periods of dryness is a characteristic feature of *A. baumannii*, contributing to its persistence in clinical environment [25]; therefore we investigated the ability of Ab_169_ and its OmpA mutants to survive under desiccation conditions. For this purpose, *A. baumannii* strains were challenged to the stress of water limitation by incubating dried bacteria on the plastic surface at 28 °C for 24 h, as described in the Materials and Methods section. The Δ*ompA* mutant was unable to survive under desiccation conditions when compared with the parent strain (Figure 2B), while the resistance phenotype was restored by the complementation with p*ompA* , although not to the full extent. Interestingly, the *ompA*_D268A_ complementation did not support the resistance phenotype of the Δ*ompA* mutant, indicating that the attachment of OmpA to the peptidoglycan contributes to *A. baumannii* resistance to desiccation stress.

### 2.3. Effect of OmpA D268A Substitution on A. baumannii Adhesion to Lung Epithelial Cells and Resistance to Serum-Mediated Killing

**Figure 3:**
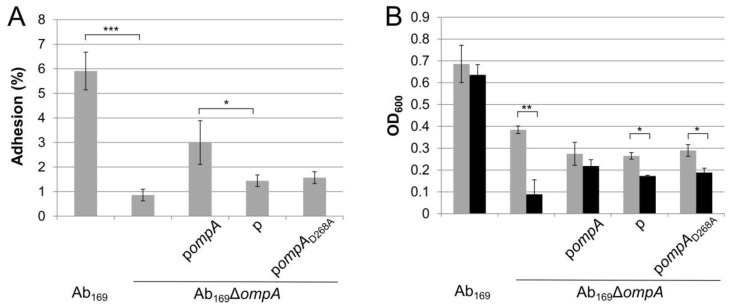
*A. baumannii* adhesion to LL/2 cells and resistance to serum. (**A**) bacterial adhesion to the LL/2 cells was expressed as a percentage of the colony-forming units (CFUs) of adhered bacteria compared with the total number of CFUs of the initial inoculum; error bars represent standard errors from at least three independent experiments. (**B**) effect of the *ompA* gene to resistance to serum-mediated killing in *A. baumannii* strains; strains were grown in LB media supplemented with 80% of heat-inactivated (grey bars) or active (black bars) fetal bovine serum (FBS) for 13 h and OD_600_ was measured; error bars represent standard deviations of three independent experiments. Significance was assessed by *t*-test (*** *p* < 0.001; ** *p* < 0.01; * *p* < 0.05).

It has been previously shown that *A. baumannii* OmpA is required for bacterial attachment to the epithelial cells [10]. Therefore, we were interested in whether the C-terminal domain of OmpA plays any role in the expression of this virulence trait. For this purpose, the Ab_169_ strain and its Δ*ompA* mutant, complemented either with an empty plasmid or with a plasmid carrying the *ompA*_D268A_ allele, were tested for the ability to adhere to the mice lung epithelium cells LL/2 (as discussed in the Materials and Methods section). As can be seen in Figure 3A, the Δ*ompA* mutant showed an approximate six-fold decrease in adhesion when compared with the parent strain, thereby confirming the role of OmpA in supporting the adhesive properties of *A. baumannii*. The *ompA* allele restored the phenotype of the Δ*ompA* mutant, although not fully. Notably, the *ompA*_D268A_ allele was deficient in its complementation ability being comparable to that of an empty plasmid (Figure 3A).

The ability to avoid host defense systems, such as complement, is a crucial feature in establishing the infection by *A. baumannii* [26]. The OmpA protein is viewed as one of the most important virulence factors involved in mediating *A. baumannii* resistance to human serum components, since the OmpA ability to bind and inactivate complement factor H has been demonstrated [11]. Therefore, we tested the capability of the Ab_169_ strain and its Δ*ompA* mutant complemented with the *ompA*_D268A_ allele and with the control plasmid to avoid serum-mediated killing. For this purpose, bacteria were grown in LB media supplemented with 80% of active or heat-inactivated fetal bovine serum (FBS). The Δ*ompA* mutant exhibited significantly reduced growth in active serum-supplemented media compared with that with the heat-inactivated serum (Figure 3B). The complementation with *ompA* restored serum resistance, whereas the presence of the *ompA*_D268A_ variant was not able to eliminate the serum sensitivity of the Δ*ompA* strain. This indicates that the OmpA protein interaction to peptidoglycan contributes to *A. baumannii* resistance to serum complement components.

### 2.4. Effect of OmpA D268A Substitution on A. baumannii Virulence in Caenorhabditis elegans Infection Model

**Figure 4:**
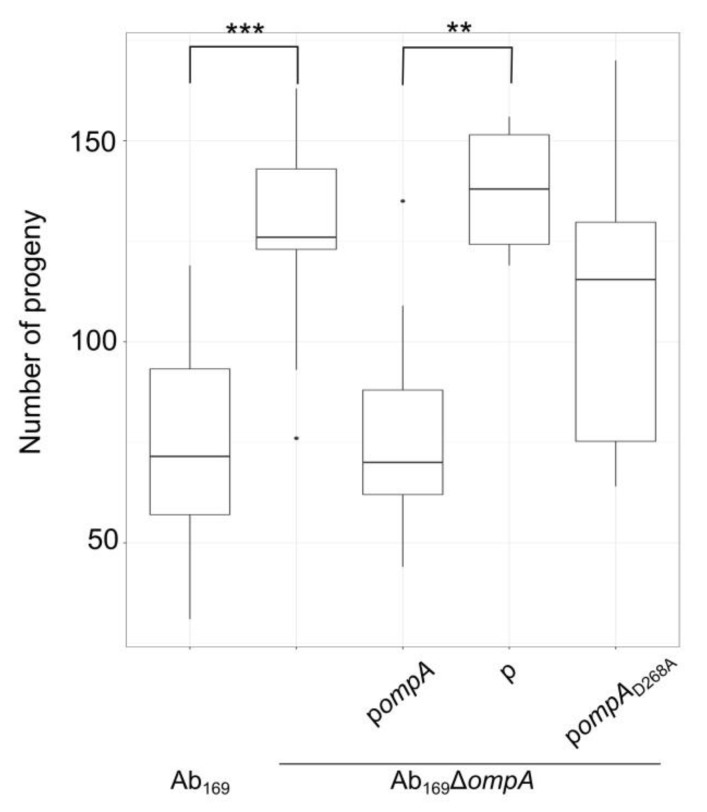
*A. baumannii* virulence in nematode infection model. *Caenorhabditis elegans* fertility was evaluated after 3 days of nematodes growth in the presence of *A. baumannii* bacteria; the box plot represents the count of nematodes progeny after incubation; data were obtained from three independent experiments, three plates were used in the each experiment; black lines represent medians and whiskers—minimum to maximum values; significance was assessed by ANOVA (*** *p* < 0.001; ** *p* < 0.01).

For the validation of the effect of D268A substitution in OmpA on *A. baumannii* virulence in vivo, we have assessed nematodes (*C. elegans*) fertility by counting worm progeny after three days upon *A. baumannii* infection (as discussed in the Materials and Methods section). We observed that *ompA* deletion impaired the virulence of the Ab_169_ strain, which was fully complemented by the *ompA* gene supplied *in trans* (Figure 4). However, the *ompA* allele with D268A substitution did not rescue the phenotype, indicating the importance of association of OmpA to peptidoglycan on *A. baumannii* infection in vivo.

### 2.5. The OmpA Association to Peptidoglycan Is Dispensable for Ampicillin Inactivation by Outer Membrane Vesicles

**Figure 5:**
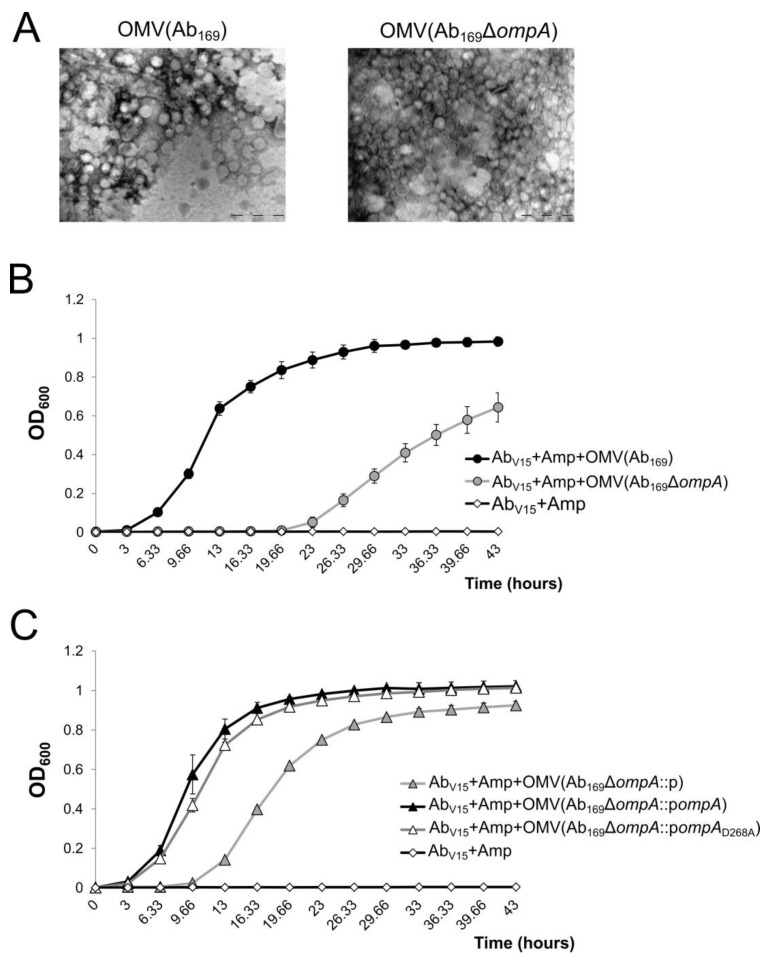
Neutralization of ampicillin by *A. baumannii* outer membrane vesicles. (**A**) visualization of outer membrane vesicles (OMVs) by transmission electron microscopy; scale bar is 0.2 µm. (**B**) growth of ampicillin sensitive *A. baumannii* strain Ab_V15_ in LB media supplemented with 50 µg/mL of ampicillin in the presence of 1 µg/mL of OMVs purified from Ab_169_ and Ab_169_Δ*ompA* strains. (**C**) growth of *A. baumannii* strain Ab_V15_ in LB media supplemented with ampicillin in the presence of OMVs purified from Ab_169_Δ*ompA* complemented strains. Error bars represent standard deviations from three repeats.

Many bacterial pathogens produce outer membrane vesicles (OMVs), which play a role in resistance to antibiotics, transport of virulence factors and other pathogenesis-related bacterial processes [27]. It has been shown that the OmpA protein is a major component of *A. baumannii* OMVs [16]. Recently, a role of selective β-lactam porin has been proposed for the OmpA protein [14]. Therefore, we wondered whether the association of OmpA with the peptidoglycan is involved in its function as a porin, transferring ampicillin into OMVs. To investigate this property, we isolated the OMVs from the Ab_169_ strain, which is characterized by a MDR phenotype and its Δ*ompA* mutant (Figure 5A). Additionally, OMVs were isolated from Ab_169_Δ*ompA* strains complemented with the *ompA*_D268A_ allele or with a control plasmid. The presence of active β-lactamases in purified OMV samples was confirmed with nitrocefin as the specific β-lactamase substrate (data not shown). Then we assessed the ability of purified OMVs supplied into the media with 50 µg/mL of ampicillin to rescue the growth of ampicillin sensitive *A. baumannii* strain Ab_V15_. As can be seen in Figure 5B, the presence of 1 µg/mL of OMVs purified from the Ab_169_Δ*ompA* mutant resulted in the growth delay of the Ab_V15_ strain compared with the presence of OMVs derived from the parent strain. OMVs from the Ab_169_Δ*ompA* strain, complemented with p*ompA*, were able to neutralize the ampicillin more efficiently, compared with the Ab_169_Δ*ompA* strain carrying the empty vector, thereby allowing the early growth of the target Ab_V15_ strain (Figure 5C). The rescue of growth of the Ab_V15_ strain was also observed with the OMVs, obtained from the Ab_169_Δ*ompA* strain complemented with the *ompA*_D268A_ allele, indicating that OmpA association with peptidoglycan is dispensable for the ability of *A. baumannii* OMVs to neutralize ampicillin.

## 3. Discussion

The *A. baumannii* OmpA is an abundant outer membrane protein, which is common for Gram-negative bacteria, including pathogenic species [28]. It is a member of a large family of homologous proteins and has been implicated in various cellular activities including bacterial adhesion, host cell invasion, immune evasion or stimulation of pro-inflammatory cytokine production [28,29,30]. The membrane domain of OmpA resembles the β-barrel, whereas the periplasmic C-terminal domain is globular and belongs to the OmpA-like domain family [15]. It has been shown that loops 1, 2 and 3 in the β-barrel domain of OmpA contribute to the binding and invasion of enteropatogenic *E. coli* into human epithelium cells [30]. The extracellular loops of *Escherichia coli* OmpA were demonstrated to bind complement regulator protein C4bp, thereby contributing to the serum resistance [31]. The β-barrel domain of *A. baumannii* OmpA has been suggested to function as antibiotic permeant porin [8,14].

The C-terminal periplasmic domain of OmpA specifically binds to the peptidoglycan via non-covalent interactions [15,17]. Two absolutely conservative D271 and R286 amino acids of the C-terminal domain *A. baumannii* OmpA have been shown to interact with diaminopimelic acid of peptidoglycan [15,17]. The interaction between the periplasmic domain of OmpA and peptidoglycan suggests that OmpA plays a role in the integrity of the bacterial surface [15]. Moreover, in a number of Gram-negative bacteria—including *A. baumannii*—the OmpA dimerization via its C-terminus has been demonstrated [13,32,33]. It is thought that dimerization stabilizes the overall structure of OmpA by preventing the collapse of the flexible linker between the β-barrel and periplasmic domains [34]. The ligand-induced conformational changes in the periplasmic domain of *A. baumannii* OmpA was reported [35]. The mechanism of OmpA interaction to peptidoglycan was proposed, which enables the stabilization of the peptidoglycan-outer membrane structure [15]. On the basis of this mechanism, a monomeric OmpA protein could cause local cell wall destruction by approximating peptidoglycan to the outer membrane, while dimeric OmpA being in contact with the peptidoglycan does not induce such perturbations. Consequently, the OmpA-association to peptidoglycan should largely contribute to the cell wall integrity. Indeed, our observations, presented in this study, show that mutation of a conservative residue involved in the interaction of OmpA with peptidoglycan-impaired cell wall integrity-dependent features, such as resistance to desiccation or serum, induced killing. Moreover, results obtained from a nematode infection assay demonstrated mixed virulent phenotypes in the population of the D268A substituted strain, as the number of nematode progeny was intermediate between the parent strain and the OmpA mutant. This could be caused by the unequal stability of the outer membrane structure in the individual bacteria. It is worth mentioning that in our study the Δ*ompA* mutant demonstrated reduced growth compared with the parent strain (Figure 3). This is in line with the observations of multiple studies investigating other bacterial pathogens, where the loss of outer membrane proteins, such as the Tol-Pal system or OmpA-like proteins, resulted in the reduced membrane integrity and alterations in cell division [36,37]. It is suggested that bacterial OmpA-like proteins might reinforce the interaction between outer membrane and peptidoglycan, typically maintained by the Tol-Pal system and Lpp lipoproteins [38]. While the exact molecular mechanisms of bacterial wall division remain largely unknown [39], the polarization of the OmpA-like protein in the outer membrane was observed during the cellular division in *Caulobacter crescentus*, implying the additional OmpA role in the multiplication of bacterial cells [40]. Our results are in line with these observations showing defects in cell division of the *A. baumannii* Δ*ompA* mutant manifested by filamentous bacterial chains observed by CLSM. The functional D268 residue of the OmpA-like domain was required for the restoration of the phenotype, thereby suggesting that the OmpA interaction with peptidoglycan is required for cell wall functioning during cell division.

Numerous studies have demonstrated the multifunctional role of the OmpA protein in *A. baumannii* pathogenesis, especially in biofilm formation and in association with epithelial cells or to the components of the immune-defense system [9,10,11]. Here, we demonstrated that a plethora of *A. baumannii* phenotypic defects, including twitching motility, resistance to desiccation, serum-mediated killing, adherence to mice lung epithelial cells and virulence in a nematode infection model, were dependent on the altered OmpA association to peptidoglycan. Several studies implicated *A. baumannii* OmpA as a principal virulence factor causing the death of epithelial cells [9,41] through the induction of caspase activation [42]. Moreover, nuclear and mitochondrial localization of OmpA in epithelial cells during *A. baumannii* infection was reported [9,41]. Our observations raise a question, whether some of the previously observed Δ*ompA* mutant phenotypes were due to the defects in cell wall integrity; therefore, additional investigations in elucidating the exact mechanism of OmpA action are needed.

Instability of the bacterial cell wall, caused by alterations in peptidoglycan synthesis, envelope stability or lipopolysaccharide synthesis, has been shown to increase a production of OMVs in bacterial pathogens such as *Salmonella enterica* [43]. The increased production of OMVs by the *A. baumannii ompA* deletion mutant was demonstrated [16], what supports the OmpA role in the maintenance of the cell wall integrity. Our results, using a clinical *A. baumannii* strain Ab_169_, are consistent with these data, since the generated *ompA* deletion mutant produced approximately 2.5-fold more OMVs than the parent strain. Numerous Gram-negative bacteria produce OMVs, which are enriched in host invasion-related effectors or proteins, which neutralize antibiotics, such as β-lactamases [44]. The production of OMVs increases when antibiotics are present in the growth media [45] and the ability of *E. coli* OMVs to inactivate β-lactams have been demonstrated [46]. The C-terminus of *A. baumannii* OmpA protein has been shown to be important for the resistance to antibiotics such as aztreonam, ciprofloxacin, colistin, gentamicin, trimethoprim and imipenem [18]. Multiple *A. baumannii* OmpA interaction sites to carbapenemase OXA-23 were identified [47], indicating its role in antimicrobial resistance. Our results argue that at least OmpA association to peptidoglycan is not necessary for OmpA-mediated resistance to ampicillin, since OMVs derived by the strain producing OmpA_D268A_ were able to efficiently neutralize the drug. This suggests that the N-terminus transmembrane β-barrel domain is an essential part of the OmpA protein for the transport of antibiotics into OMVs, and that association to peptidoglycan is not required for this process. Additionally, our study provides a suitable model for the investigation of porin-mediated compound transferring into OMVs.

## 4. Materials and Methods

### 4.1. Bacterial Strains and Growth Conditions

Strains used in this study are listed in the Table S1. Bacteria were grown on Luria–Bertani (LB) plates at 37 °C. Liquid cultures were grown in LB medium overnight.

### 4.2. Motility Assays

Twitching and swarming motilities were investigated as previously described [24]. Motility was quantified by measuring the halo of growth around the inoculation.

### 4.3. Desiccation Assay

*A. baumannii* strains were tested for their ability to survive under desiccation stress as described by [24]. Pre-desiccated samples were serially diluted into 10-fold dilutions and seeded on the LB plates. After desiccation, samples were resuspended in LB broth, serially diluted into 10-fold dilutions and seeded.

### 4.4. Confocal Laser Scanning Microscopy (CLSM)

For evaluation of biofilms, 1000-fold dilutions of overnight *A. baumannii* cultures were used for seeding into LB media. Biofilms were grown for 2 h and 24 h at 37 °C without agitation. After growth in micro-titer plates (µclear Greiner BioOne, Les Ulis, France), biofilms were stained for 2 h by the Filmtracer LIVE/DEAD Biofilm Viability Kit (Thermo Fisher Scientific, Les Ulis, France). The plate was then placed on the motorized stage of an inverted confocal microscope (TCS SP8 AOBS, Leica Microsystems, Nanterre, France) at the INRA-MIMA2 imaging platform and results were analyzed as described previously [48].

### 4.5. Isolation of Outer Membrane Vesicles

Bacteria were grown in 130 mL of LB broth for 20 h at 37 °C with 145 rpm shaking. Bacterial cells were removed via 15 min of centrifugation at 10,000× *g* at 4 °C and filtration through a 0.22 µm filter (Sigma-Aldrich, St. Louis, MO, USA). The OMVs were collected via 3 h of ultracentrifugation at 130,000× *g* at 4 °C. OMV pellets were resuspended in 0.11 mL phosphate-buffered saline (PBS). The protein concentration was determined using Bradford assay (Roth). 10 µL of OMV solution was plated on LB agar to test the sterility. The purified OMVs were stored at −20 °C.

### 4.6. Transmission Electron Microscopy (TEM)

TEM analysis was undertaken at the Institute of Biotechnology (Vilnius University, Lithuania). OMV samples were placed on a 300-mesh carbon coated palladium grid, negatively stained with 2% aqueous uranyl acetate solution and examined by a Morgagni 268 electron microscope (FEI Inc., Hillsboro, OR, USA).

### 4.7. A. baumannii Growth Assays

Bacterial growth was evaluated in LB medium, 80% active fetal bovine serum (FBS) and 80% heat inactivated FBS (htFBS) as described by [24]. For OMV assays, LB media with 50 µg/mL of ampicillin in the presence of 1 µg/mL of OMV purified from Ab_169_ strains were incubated at 37 °C for 15 min. Then, the ampicillin sensitive *A. baumannii* strain Ab_V15_ was grown in pre-incubated LB media. After growing the Ab_V15_ strain with OMVs, liquid cultures were seeded on LB agar plates and supplemented with 50 µg/mL of ampicillin to deny the contamination or simultaneous mutations in Ab_V15_. Growth was measured at 37 °C with periodic shaking every 20 min by a Tecan Infinite M200 Pro microplate reader.

### 4.8. Generation of ΔompA Deletion Mutant, Complemented Strains and Site-Directed Mutagenesis

Markerless gene deletion was performed as described previously [49]. Plasmids used for mutant generation are listed in Table S1. Mutants were selected by PCR with specific primers and confirmed by sequencing. For the complementation, the sequence of the *ompA* gene with upstream region (with the putative native promoter sequences) was amplified using primers listed in Table S1 and cloned into the pUC19_gm_AcORI plasmid. Site-directed mutagenesis of the OmpA-like domain was performed using inverse PCR with primers OAsp268F/OAsp268R and the p*ompA* plasmid as a template (Table S1). All generated plasmids were confirmed by sequencing. The *ompA* gene deletion mutant was transformed with the resulting plasmids by electroporation and colonies were selected on LB agar with 30 μg/mL of gentamicin.

### 4.9. SDS-PAGE and Immunoassay

For protein analysis, cultures were grown in LB media at 37 °C overnight, suspended in PBS, normalized to an OD_600_ = 0.5 and lysed by sonication. Fractions of membranous proteins were separated by ultracentrifugation. Bacterial cells were suspended in 10 mM Tris base (pH 7.5) and frozen at −80 °C for 2 h. Samples were sonicated and centrifuged at 7000× *g* for 10 min to remove cell debris. Supernatants were proceeded by ultracentrifugation at 120,000× *g* for 45 min at 4 °C. The insoluble materials were suspended in PBS. Samples were loaded on SDS-PAGE gels as previously described [24] and stained with Coomassie Blue.

For Western blot analysis, proteins were transferred on nitrocellulose membrane (Amersham Biosciences, Pittsburgh, PA, USA) using the semi-dry method [50]. As primary antibodies, 1:5000 dilutions of OmpA-specific serum obtained from immunized mice was used. After this, the membrane was exposed to goat anti-mouse IgG (H + L)-HRP conjugate (Bio-Rad, Hercules, CA, USA) developed with pierce one-step ultra TMB blotting solution (ThermoFisher Scientific, Walkersville, MD, USA).

### 4.10. Cell Culture Assays

Mouse epithelial LL/2 (LLC1) cells [24] were grown in Dulbecco’s modified Eagle’s medium (DMEM) supplemented with 10% FBS at 37 °C with 5% CO_2_. Adhesion experiments were performed as described by [24]. Lung epithelial cells were plated at a density of 1.5 × 10^4^ cells/well into 96-well tissue culture plates. Cells were grown for 48 h to form a fastened culture monolayer with ~80% confluence. LL/2 cells were infected with bacteria at a multiplicity of infection (MOI, bacteria: eukaryotic cell ratio) ~1000:1. Serially diluted cells lysates were plated onto LB medium to determine the number of adhered bacteria. Bacterial adherence to the LL/2 cells was expressed as a percentage of the CFU of adhered bacteria compared with the total number of CFUs of the initial inoculum.

### 4.11. Caenorhabditis elegans Fertility Assay

*A. baumannii* strains were investigated using the *C. elegans* fertility model as described by [24]. Overnight cultures of different *A. baumannii* strains were seeded on a nematode growth medium (NGM). One L2 stage worm was placed over each *A. baumannii* strain. On the third day after infection, the worm progeny was determined by counting *C. elegans* worms.

### 4.12. Statistical Analysis

All statistical comparisons were based on *t*-test or the one-way analysis of variance (ANOVA) with a Tukey HSD (honestly significant difference) post hoc test. All quantitative data are representative of at least three repeats.

## 5. Conclusions

Overall, we demonstrate in this study that the interaction between OmpA and peptidoglycan is an essential condition for the functioning of OmpA protein in *A. baumannii*, including virulent properties of bacteria. The dual effect of *ompA* deletion on the formation of *A. baumannii* filamentous bacterial chains and cell wall permeability was established.

## Supplementary Materials

The following are available online. Figure S1: Protein profiles and Western blotting with anti-OmpA serum of bacterial lysate samples fractionated by 12% SDS–PAGE; Table S1: Oligonucleotides, plasmids and strains used in this work.
