# Supplementary material for: The Mutation of Conservative Asp268 Residue in the Peptidoglycan-Associated Domain of the OmpA Protein Affects Multiple Acinetobacter baumannii Virulence Characteristics
Source: Molecules. 2019 May 22;24(10):1972. doi: 10.3390/molecules24101972 (PMC6572160; doi:10.3390/molecules24101972)
Supplement: Supplementary file 1 [file molecules-24-01972-s001.zip › Supplementary/Figure_S1.pdf]

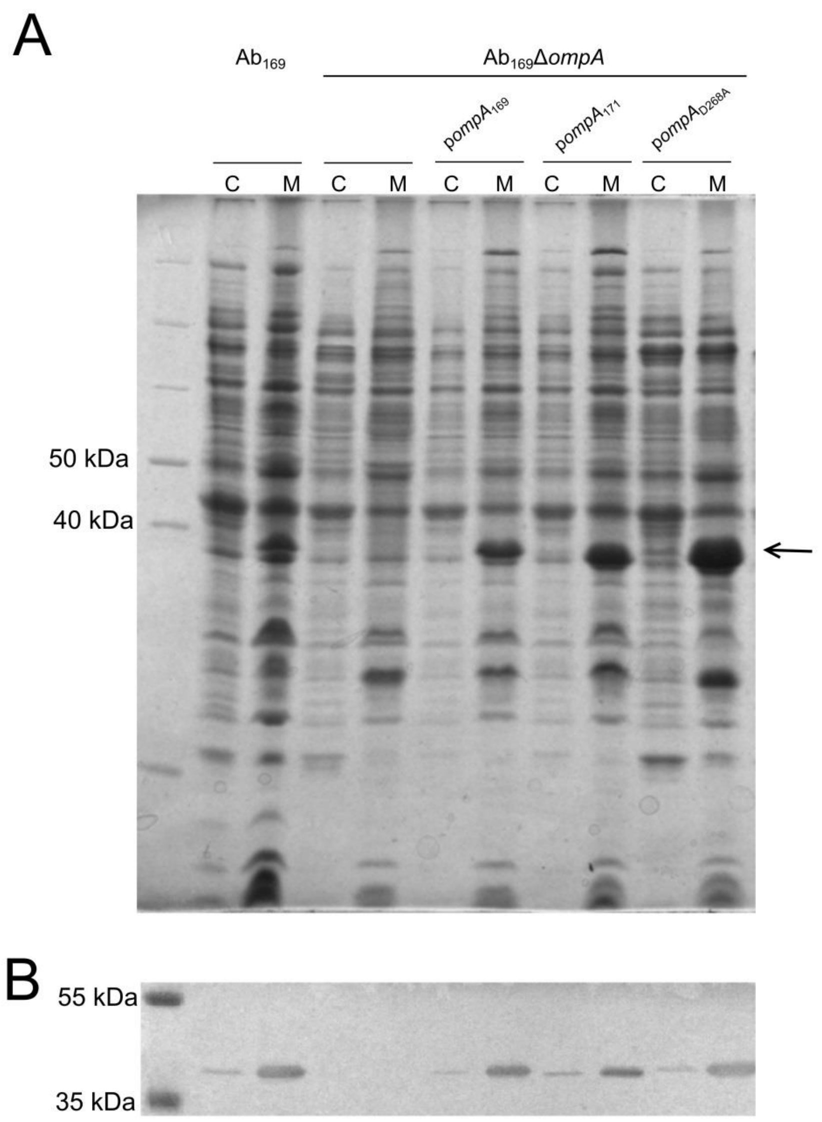

Figure S1. Protein profiles and Western blotting with anti-OmpA serum of bacterial lysate samples fractionated by 12% SDS –PAGE. A – cytosolic (C) and membranous (M) proteins fractions, extracted from A. baumannii strains were loaded on SDS-PAGE gel; additional complementation strain with ompA gene allele from Ab171 strain, belonging to IC II, was added; arrow indicates OmpA protein. B – Wester-Blot analysis using OmpA specific serum obtained from immunized mice. The positions of standard molecular mass markers (PageRuler™ Prestained Protein Ladder (Thermo Fisher Scientific)) are shown on the left.
