# Supplementary material for: The Mutation of Conservative Asp268 Residue in the Peptidoglycan-Associated Domain of the OmpA Protein Affects Multiple Acinetobacter baumannii Virulence Characteristics
Source: Molecules. 2019 May 22;24(10):1972. doi: 10.3390/molecules24101972 (PMC6572160; doi:10.3390/molecules24101972)
Supplement: Supplementary file 1 [file molecules-24-01972-s001.zip › Supplementary/Table_S1.pdf]

Table S1. Oligonucleotides, plasmids and strains used in this work.

Primers	Sequence (5'to 3')	Purpose	Source
Fjgms	AGGACAGAAATGCCTCGAC	*aac(3)-I* amplification	[1]
Rjgms	ATCTCGGCTTGAACGAATT		
OmpA1	GGAGCAGTTAGTCCTGATAG	*ompA* upstream region	This work
OmpA2	GAACTCAAATTATTGAGCTGCCTCCAGAGATAACAATTG	amplification; additional sequence underlined	
OmpA3	CAGCTCAATAATTTGAGTTC	*ompA* downstream region	This work
OmpA4	GTCGAGGCATTTCTGTCCTTGCTTCGTCAGTTTGAGGC	amplification; additional sequence underlined	
OmpAP	GCGATTGCTTCTTTAATTGT	*ompA* gene deletion confirmation with OmpA4 primer	This work
OmklF	CCTTAATATATGTATAAATAGAGC	*ompA* gene with upstream region	This work
OmklR2	AGTCGGTACCAGATTATGAATCAGGAGATTTAC	amplification	
Aac3I_seqR	CGAAGTCGAGGCATTTCTGT	Confirmation of transformed	This work
AcORI_seqR	AGGCTGTTGATAACTTTTGGAA	bacteria	
OAsp268F	CACACAGCTAACACTGGTCCACGTAAG	Asp268 substitution by Ala in	This work
OAsp268R	GACCAGTGTTAGCTGTGTGACCTTCG	*ompA* gene	

Plasmids	Relevant characteristics	Source
pUC19_sacB	pUC19 derivative with *sacB* gene from *Bacillus* sp.; for the generation of markerless gene deletion mutants	[1]
pUC19_gm_AcORI (p)	pUC19 derivative with *aac(3)-I* gentamicin aminoglycoside acetyltransferase cassette and ori site from clinical *A. baumanni* strain; for the complementation experiments	This work
pUC_sacB_UDompAGm	pUC19_sacB derivative with upstream and downstream regions of *A. baumannii ompA* gene and *aac(3)-I* gentamicin aminoglycoside acetyltransferase cassette from clinical *A. baumanni* strain; for the generation of markerless gene deletion mutant	This work
p*ompA*	pUC19_gm_AcORI derivative with *ompA* gene along with upstream region (plausible promoter) from Ab_{169} strain; for the complementation experiments	This work

p*ompA*$_{171}$	pUC19_gm_AcORI derivative with *ompA* gene along with upstream region (plausible promoter) from Ab$_{171}$ strain; for the complementation experiments	This work
p*ompA*$_{D268A}$	pUC19_gm_AcORI derivative with *ompA* gene with D268A substitution along with upstream region (plausible promoter) from Ab$_{169}$ strain; for the complementation experiments	This work
**Strains**	**Relevant characteristics**	**Source**
Acinetobacter baumannii Ab$_{169}$	Representative IC I clone strain[a]; MDR strain, gentamicin sensitive	[2]
Acinetobacter baumannii Ab$_{171}$	Representative IC II clone strain[a]; MDR strain, gentamicin sensitive	[2]
Acinetobacter baumannii Ab$_{V15}$	Non-clonal strain[a]; ampicillin sensitive	[2]
Ab$_{169}$*ΔompA*	*ompA* gene-negative mutant of *A. baumannii* strain Ab$_{169}$; markerless	This work
Ab$_{169}$*ΔompA*::p	Ab$_{169}$*ΔompA* strain with pUC19_gm_AcORI plasmid	This work
Ab$_{169}$*ΔompA::*p*ompA*	Ab$_{169}$*ΔompA* strain complemented with p*ompA*	This work
Ab$_{169}$*ΔompA::*p*ompA*$_{171}$	Ab$_{169}$*ΔompA* strain complemented with p*ompA*$_{171}$	This work
Ab$_{169}$*ΔompA::*p*ompA*$_{D268A}$	Ab$_{169}$*ΔompA* strain complemented with p*ompA*$_{D268A}$	This work
Ab$_{IC\ II}$*Δblp1::*p*blp1*$_{IC\ II}$	Ab$_{IC\ II}$ *Δblp1* strain complemented with p*blp1*$_{IC\ II}$	This work
Escherichia coli OP50	Wild type, bacterial food source for *C. elegans*	[3]
E. coli JM107	endA1 glnV44 thi-1 relA1 gyrA96 Δ(lac-proAB) [F_ traD36 proAB+ lacIq lacZΔM15] hsdR17(RK- mK+) λ-; selection of constructed vectors	[4]

[a] Strains were assigned to IC I and IC II by trilocus sequence-based typing (3LST) as described previously (Povilonis et al., 2013); Strains were isolated from Lithuanian University of Health Sciences Kauno Klinikos Hospital or Vilnius University Emergency Hospital in 2010.

[1] Skerniškytė J, Krasauskas R, Péchoux C, Kulakauskas S, Armalytė J, Sužiedėlienė E. Surface-related features and virulence among *Acinetobacter baumannii* clinical isolates belonging to international clones I and II. Front. Microbiol. 2019;9:3116.

[2] Povilonis J, Seputiene V, Krasauskas R, Juskaite R, Miskinyte M, Suziedelis K, et al. Spread of carbapenem-resistant *Acinetobacter baumannii* carrying a plasmid with two genes encoding OXA-72 carbapenemase in Lithuanian hospitals. J Antimicrob Chemother. 2013;68(5):1000-6.

[3] Brenner S. The genetics of *Caenorhabditis elegans*. Genetic. 1974; 77: 71–94;

[4] Yanisch-Perron C, Vieira J, Messing J. Improved M13 phage cloning vectors and host strains: nucleotide sequences of the M13mp18 and pUC19 vectors. Gene. 1985;33(1):103-19. Erratum in: Gene. 1992;114(1):81-3.
